# The influence of actin depolymerization induced by Cytochalasin D and mechanical stretch on interleukin-8 expression and JNK phosphorylation levels in human retinal pigment epithelial cells

**DOI:** 10.1186/s12886-017-0437-z

**Published:** 2017-04-07

**Authors:** Meng Gao, Shen Wu, Jing Ji, JingXue Zhang, Qian Liu, YanKun Yue, Lu Liu, XinXin Liu, Wu Liu

**Affiliations:** 1 Beijing Tongren Eye Center, Beijing Tongren Hospital, Capital Medical University; Beijing Ophthalmology and Visual Sciences Key Laboratory, Beijing, China; 2 Beijing Institute of Ophthalmology, Beijing Tongren Eye Center, Beijing Tongren Hospital, Capital Medical University; Beijing Ophthalmology and Visual Sciences Key Laboratory, Beijing, China; 3 Key Laboratory for Biomechanics and Mechanobiology of Ministry of Education, School of Biological Science and Medical Engineering, Beihang University, Beijing, China; 4 Department of Ophthalmology, Beijing Fuxing Hospital, Capital Medical University, Beijing, China; 5 Department of Ophthalmology, Kailuan General Hospital, Tangshan, China

**Keywords:** Stress, Mechanical, Retinal pigment epithelium, Cytoskeleton, Cytokines, Cytochalasin, JNK Mitogen-activated protein Kinases

## Abstract

**Background:**

This study explores the role of actin cytoskeleton depolymerization induced by Cytochalasin D and mechanical stretch on the interleukin-8 (IL-8) expression and c-jun N-terminal kinase (JNK) phosphorylation levels in human retinal pigment epithelial (RPE) cells.

**Methods:**

A Flexcell FX-5000 Tension system was used to apply cyclic stretch to cultured human RPE cells (ARPE-19) at 0.33 Hz with 20% elongation for 0 h, 6 h or 24 h. The cells were stretched alone or pre-treated with Cytochalasin D. The redistribution of the actin cytoskeleton was evaluated using phalloidin immunofluorescence staining. The protein expression levels of IL-8 and JNK in the RPE cells were determined via Western blotting.

**Results:**

The cells in the control groups displayed abundant and uniform phalloidin staining. After exposure to mechanical stretch for 24 h, phalloidin staining revealed an unclear and irregular actin cytoskeleton. In all Cytochalasin D-treated cells, the shrinkage and disruption of the cytoskeletal structure was observed regardless of mechanical stress. The stimulation of the RPE cells with cyclic stretch alone did not induce a significant increase in IL-8 expression and JNK phosphorylation levels, which were similar to those of the control groups. After pre-treatment with Cytochalasin D alone, IL-8 expression and JNK phosphorylation levels were not significantly different at 6 h but were significantly increased by approximately 1.2-fold (1.18 ± 0.05; *P*<0.01) and 3.0-fold (3.01 ± 0.02; *P*<0.01) at 24 h, respectively. After the pre-incubation of the RPE cells with Cytochalasin D followed by exposure to cyclic stretch, IL-8 expression and JNK phosphorylation levels increased by approximately 1.3-fold (1.31 ± 0.02; *P*<0.01) and 1.3-fold (1.31 ± 0.02; *P*<0.01) at 6 h, respectively, and by 1.7-fold (1.69 ± 0.06; *P*<0.01) and 3.2-fold (3.21 ± 0.12; *P*<0.01) at 24 h, respectively.

**Conclusions:**

This study demonstrates that disruption of actin polymerization by cytochalasin D and mechanical stretch upregulates interleukin-8 expression and JNK phosphorylation levels in human RPE cells, which indicates that the integrity of the actin cytoskeleton may play important roles in the pro-inflammatory processes in RPE cells.

## Background

The retinal pigment epithelium (RPE) is a monolayer of pigment-containing epithelial cells in the retina, which plays a major role in normal retinal function and the pathogenesis of retinal diseases. RPE cells effectively secrete interleukin-8 (IL-8) in response to tumour necrosis factor (TNF)-α, C-reactive protein, C5a and other pro-inflammatory stimuli [1–4]. Several lines of evidence have indicated that high levels of IL-8 in the aqueous humour and vitreous fluid are significantly associated with the activity of various vitreoretinal diseases, such as age-related macular degeneration, diabetic macular oedema, proliferative diabetic retinopathy and rhegmatogenous retinal detachment [5–7]. Exploring the mechanism by which RPE cells secrete IL-8 contributes to the understanding of the aetiology of these retinal diseases and informs treatment.

Mechanical stimulations play critical roles in the physiological functions and pathological processes of living cells. Published data have demonstrated that RPE cells experience chronic mechanical stress transmitted from the vitreoretinal interface and the neural retina [8, 9]. This mechanical stress may upregulate the expression levels of several pathological factors in RPE cells, such as vascular endothelial growth factor and metalloproteinases [10, 11]. However, little is known about the expression of interleukins in RPE cells under mechanical stress, and the precise underlying mechanisms remain unclear.

The use of Cytochalasin D, a specific inhibitor of actin polymerization, has demonstrated that actin cytoskeleton depolymerization may play an important role in mechanical stress-induced interleukin expression and c-jun N-terminal kinase (JNK) phosphorylation levels in various types of cells [12–14]. However, whether Cytochalasin D-induced actin depolymerization facilitates or attenuates stress-induced IL-8 expression and JNK phosphorylation levels is still unclear. To address this issue, we tested the effects of Cytochalasin D and mechanical stretch, which alter the actin cytoskeleton, on the IL-8 expression and JNK phosphorylation levels in cultured RPE cells. Here, we present evidence that the disruption of actin polymerization by Cytochalasin D and mechanical stretch upregulates the IL-8 expression and JNK phosphorylation levels in RPE cells in vitro.

## Methods

### RPE cell culture

ARPE-19 cells were kindly provided by Dr. Shen Wu (Beijing Tongren Hospital, China) who purchased the cells from the American Type Culture Collection. The cells were routinely cultured in Dulbecco’s Modified Eagle Medium/Ham’s Nutrient Mixture F12 (1:1) (DMEM/F12, Gibco, Grand Island, NY) supplemented with 10% foetal bovine serum (FBS, Gibco, Australia). Then, the cells were incubated at 37 °C in a humidified incubator under 5% CO_2_. The medium was changed every two to three days. The cells were trypsinized for 1 min with a 0.25% trypsin/EDTA (Gibco) solution and subcultured at a split ratio of 1:3–5 in a 25-mm^2^ plastic bottle (Corning Ltd., Lowell, MA) in an incubator to achieve a heavy primary monolayer.

### Cyclic stretch experiments

Equal amounts of cells were seeded in each well in a 6-well Collagen I-coated Bioflex culture plate (Flexcell International Corporation, Burlington, CA, USA) and grown to approximately 80% confluence. These plates were then transferred to the base plate of the cell stretching equipment, a Flexcell FX-5000TM Tension System (Flexcell International Corporation, Burlington, CA, USA), in a humidified incubator at 37 °C under 5% CO2. The cells were subjected to cyclic mechanical stretch with the following parameters: a stretching rate of 20% with a half sine signal, 0.33 Hz frequency (20 cycles/min) and a 1:1 stretch:relaxation ratio. The cells were stretched for 6 h or 24 h as described in the results. The control Bioflex plates were maintained in the same incubator under static conditions as non-stretch controls. The Cytochalasin D (ThermoFisher, USA) (2 μM) pre-treatment was applied for 30 min to cause the disruption of the actin filaments and the inhibition of actin polymerization.

### Immunofluorescence staining

The samples were collected and fixed with 4% paraformaldehyde for 30 min, followed by permeabilization in PBS with 0.3% Triton X-100 for 15 min at room temperature. The cells were then incubated with Rhodamine phalloidin for 1 h at 37 °C and washed three times with PBS and mounted with a Gold Antifade Mount with DAPI (ThermoFisher, USA). The cells were analysed using a confocal laser scanning microscope (Leica TCS-NT, Germany). At least three independent random fields per sample were captured from three independent experiments. The acquired images were converted into binary images for the quantification of the average fluorescence signal of the F-actin fibres using ImageJ software (National Institutes of Health, USA).

### Western blotting analysis

The cells were washed three times with PBS at 4 °C and then lysed in 150 μl cold 1× RIPA lysis buffer (Cell Signaling Technology, USA) supplemented with protease inhibitor cocktail tablets (Roche, Germany). The protein concentration was determined using a BCA Protein Assay Kit (Thermo Scientific, USA). The protein extracts were separated using 15% sodium dodecyl sulfate-polyacrylamide gel electrophoresis (SDS-PAGE), blotted onto polyvinylidene difluoride (PVDF) membranes (Roche) and blocked with Tris-buffered saline with 0.05% Tween 20 (TBST) containing 5% non-fat milk. The membranes were then incubated with the following primary antibodies: rabbit polyclonal IL-8 (Abcam, USA) (1:500), rabbit polyclonal JNK (Cell Signaling, USA) (1:500), and rabbit polyclonal GAPDH (Santa Cruz, USA) (1:1000). The primary antibodies were diluted in TBST with 5% non-fat milk and incubated with the membranes overnight at 4 °C. Then, the membranes were washed with TBST and incubated with a secondary goat anti-rabbit lgG antibody (ThermoFisher, USA) conjugated to horseradish peroxidase at 1:1000 for 1 h at room temperature. Finally, the chemiluminescence signals were visualized with an ECL imager (Millipore, USA) and analysed using BIO-RAD Quantity One Imaging software (Bio-Rad, USA).

### Statistical analysis

The quantitative data are expressed as the means ± SD. The statistical significance of the inter-group differences was determined with a one-way analysis of variance (ANOVA) followed by Bonferroni’s multiple comparison test. Bartlett’s test was used to determine the homogeneity of variance. *P* values less than 0.05 were considered statistically significant.

### Ethical statement

This research did not involve live human or animal subjects. This research was carried out on the ARPE-19 cell line in vitro from the American Type Culture Collection.

## Results

### Reorganization of the actin cytoskeleton induced by cyclic stretch and Cytochalasin D

**Fig. 1:**
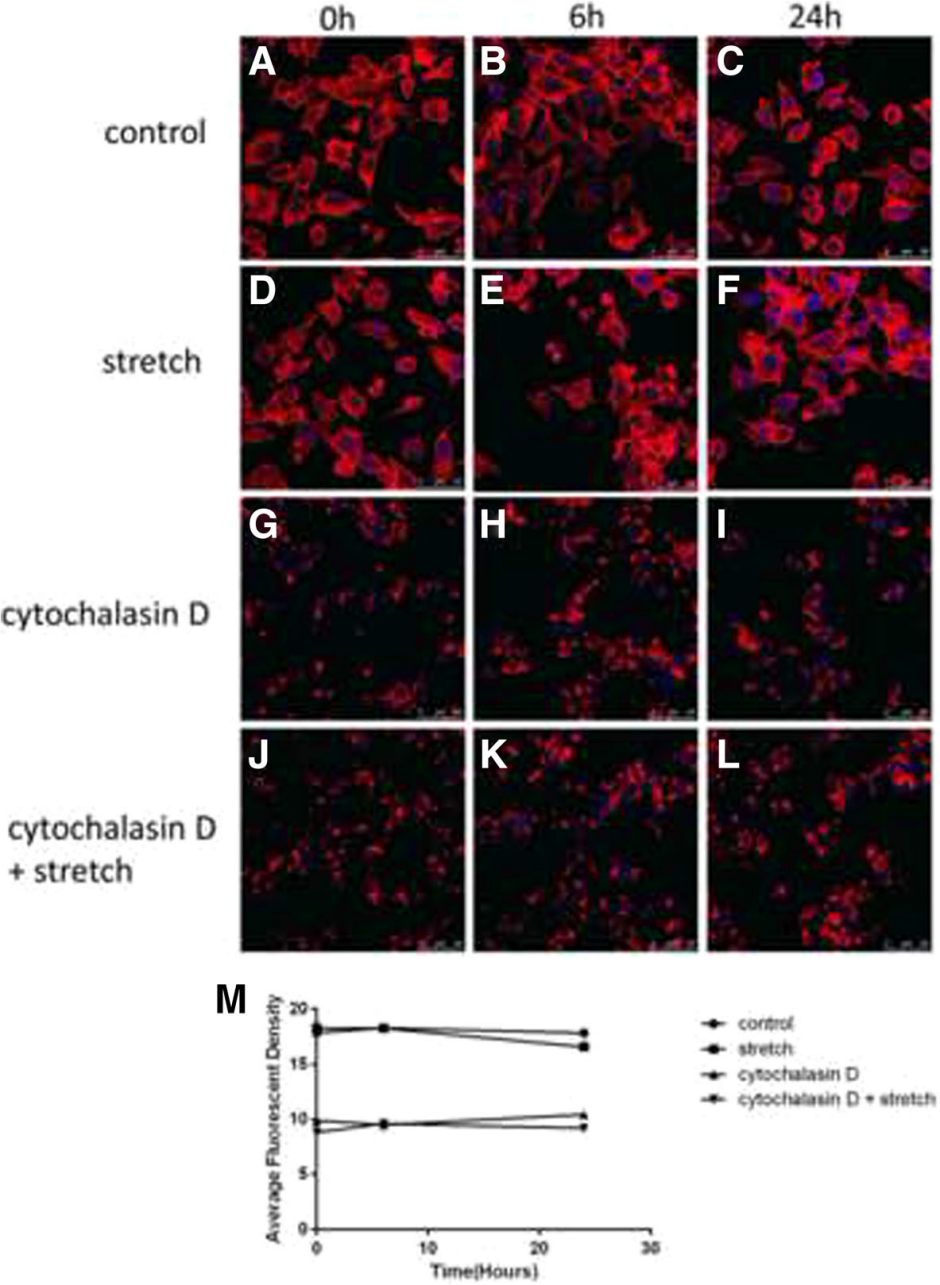
Fluorescence confocal microscopic analysis of the actin cytoskeleton in the cultured human ARPE-19 cells. The cells were subjected to mechanical stretch (20%, 0.33 Hz) for 0, 6, or 24 h with or without pre-treatment with 2 μM Cytochalasin D. The structure of the actin cytoskeleton was examined with phalloidin-Rhodamine staining. The cells in the control groups displayed abundant and uniform phalloidin staining (**a**-**c**). After exposure to mechanical stretch for 24 h, the phalloidin-stained actin cytoskeleton appeared unclear and irregular with a statistically significant decrease in the average fluorescence intensity of F-actin fibres (**d**-**f**, **m**). In all the Cytochalasin D-treated cells, the disruption of the cytoskeletal structure and a decrease in the average fluorescence intensity were observed regardless of mechanical stress (**g**-**l**, **m**). *P<0.05 versus baseline, *n* = 3 experiments

The effect of mechanical stretch and Cytochalasin D on the actin cytoskeleton was assessed via fluorescence confocal microscopy using phalloidin-Rhodamine staining on ARPE-19 cells. The cells in the control groups displayed abundant and uniform phalloidin staining (Fig. 1a-c). After exposure to mechanical stretch for 24 h, the phalloidin-stained actin cytoskeleton appeared unclear and irregular with a statistically significant decrease in the average fluorescence intensity of F-actin fibres (*P*<0.05; *n* = 3) (Fig. 1f, Fig. 1m ). In all the Cytochalasin D-treated cells, the disruption of the cytoskeletal structure and a decrease in the average fluorescence intensity were observed regardless of mechanical stress (Fig. 1g-l, Fig. 1m).

### Cytochalasin D and mechanical stretch increased IL-8 expression

**Fig. 2:**
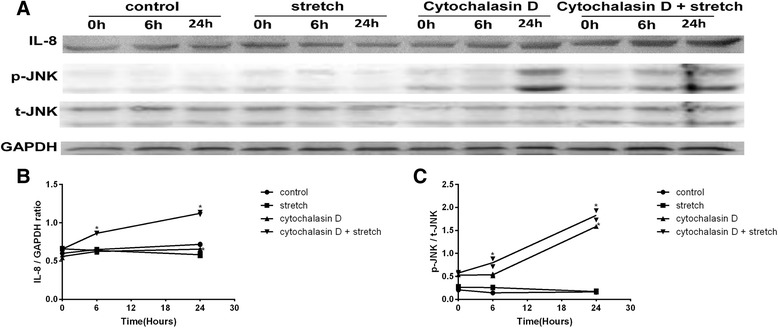
IL-8 expression and JNK phosphorylation levels in RPE cells. After cyclic stretch and Cytochalasin D exposure, cell lysates were prepared, and Western blotting analysis was performed on the indicated proteins. The IL-8 and JNK bands were scanned, and band densities were calculated using ImageJ software. The values are the band-density ratios compared with the baseline. The stimulation of the RPE cells with cyclic stretch alone did not induce a significant increase in IL-8 expression and JNK phosphorylation levels, which were similar to those of the control groups. After pre-treatment with Cytochalasin D alone, IL-8 expression and JNK phosphorylation levels were not significantly different at 6 h but were significantly increased at 24 h. After the pre-incubation of the RPE cells with Cytochalasin D followed by exposure to cyclic stretch, IL-8 expression and JNK phosphorylation levels increased at 6 h and 24 h (**a**-**c**). *P<0.01 versus baseline, *n* = 3 experiments

Cyclic stretch alone did not induce a significant increase in IL-8 expression. Treatment with Cytochalasin D alone increased IL-8 expression by approximately 1.2-fold (1.18 ± 0.05; *P*<0.01; *n* = 3) at 24 h. The pre-incubation of the RPE cells with Cytochalasin D followed by exposure to cyclic stretch increased IL-8 expression by approximately 1.3-fold (1.31 ± 0.02; *P*<0.01; *n* = 3) at 6 h and 1.7- fold (1.69 ± 0.06; *P*<0.01; *n* = 3) at 24 h (Fig. 2a, b).

### Cytochalasin D and mechanical stretch increased JNK phosphorylation levels

Cell lysates were analysed via Western blotting to measure the total protein levels and phosphorylated forms of JNK. Although the cyclic stretch treatment alone did not alter the level of phospho-JNK (p-JNK), the addition of Cytochalasin D induced JNK activation. Treatment with Cytochalasin D alone increased p-JNK expression by approximately 3.0-fold (3.01 ± 0.02; *P*<0.01; *n* = 3) at 24 h. The pre-incubation of the RPE cells with Cytochalasin D followed by exposure to cyclic stretch increased p-JNK expression by approximately 1.3-fold (1.33 ± 0.20; *P*<0.01; *n* = 3) at 6 h and 3.2-fold (3.21 ± 0.12; *P*<0.01; *n* = 3) at 24 h (Fig. 2a, b).

## Discussion

In this study, we provide evidence that the disruption of actin polymerization by Cytochalasin D and mechanical stretch enhanced IL-8 expression and JNK phosphorylation in human RPE cells, which indicates that the integrity of the actin cytoskeleton may play important roles in pro-inflammatory processes in RPE cells.

The precise mechanotransduction mechanisms by which RPE cells convert mechanical stretch stimulation into biochemical signals are still unclear. The cytoskeletal system, coupled with cell surface receptors, appears to play important roles in cellular mechanotransduction, which may transduce extracellular mechanical signals into intracellular biological responses through force-dependent changes [15]. Using beads coated with ligands or anti-integrin antibodies to trigger rapid focal transmembrane responses can induce the reorganization of the actin cytoskeleton and the rapid activation of signal transduction molecules [16]. Taijik A et al. used three-dimensional magnetic twisting cytometry to apply local stresses on the cell surface and found that stressors propagate from the tensed actin cytoskeleton to the chromatin through specific structures and ultimately upregulate transcription [17]. In the present study, we have shown that the coordination of Cytochalasin D and mechanical stretch induced actin depolymerization and pro-inflammatory processes, which indicates that the actin cytoskeleton could serve as a mechanotransducer in RPE cells.

Using a Flexcell Tension system, we found that the pre-incubation of RPE cells with Cytochalasin D followed by exposure to cyclic stretch resulted in significant increases in IL-8 expression and JNK phosphorylation levels at 6 h and 24 h compared to those induced by cyclic stretch alone and Cytochalasin D alone. There are disputes about the effects of Cytochalasin D on stress-induced IL-8 expression and JNK phosphorylation levels. Okada M et al. and Hsu HJ et al. have reported that Cytochalasin D treatment decreased stretch-induced JNK phosphorylation and IL-8 expression levels in human endothelial cells, bovine aortic endothelial cells and human osteosarcoma cells [12, 14]. Okada M et al. have hypothesized that the disruption of the actin cytoskeleton by Cytochalasin D may block the activation of the protein kinases induced by cyclic stretch, influencing interleukin expression levels. Cheng M et al. have demonstrated that Cytochalasin D increases shear stress-induced IL-8 mRNA expression levels in human endothelial cells and thought that the redistribution of the actin cytoskeleton facilitates the activation of signalling molecules [13]. This discrepancy may be due to differences in the mechanical properties, cell specificity and the Cytochalasin D treatment methods among the studies. We propose that the upregulation in our study may be due to the facilitated translocation and activation of various signalling molecules caused by the redistribution of the actin cytoskeleton in RPE cells, which requires verification by future studies.

However, our study has several limitations. The effects of actin stabilization on interleukin expression were not addressed in this study. It would be interesting to explore whether actin stabilization has a reverse effect on stress-induced interleukin expression levels. Furthermore, although ARPE-19 cells have structural and functional properties characteristic of RPE cells in vivo, the difference between the cell line and primary RPE cells cannot be ignored.

## Conclusion

In summary, our results demonstrate that the Cytochalasin D- and mechanical stretch-induced actin cytoskeleton alterations upregulate IL-8 expression and JNK phosphorylation levels in cultured RPE cells. Increased IL-8 expression in RPE cells may potentially contribute to retinal pathology processes. These findings contribute to our understanding of retinopathies since IL-8 is one of the known cytokines that play roles in the development and progression of these diseases.

## Abbreviations

ANOVA: One-way analysis of variance
ARPE-19: A spontaneously arising RPE cell line derived in 1986 by Amy Aotaki-Keen from the normal eyes of a 19-year-old male who died from head trauma in a motor vehicle accident
DMEM/F12: Dulbecco’s Modified Eagle Medium/Ham’s Nutrient Mixture F12
EDTA: Ethylenediaminetetraacetic acid
FBS: Foetal bovine serum
GAPDH: Glyceraldehyde-3-phosphate dehydrogenase
IL-8: Interleukin-8
JNK: c-jun N-terminal kinase
PBS: Phosphate-buffered saline
PVDF: Polyvinylidene difluoride
RPE: Retinal pigment epithelial
SDS-PAGE: Sodium dodecyl sulphate-polyacrylamide gel electrophoresis
TBST: Tris-buffered saline with 0.05% Tween 20
